# 

**Published:** 1887-03

**Authors:** 


### MATTERS OF INTEREST.

---

AMERICAN MEDICAL ASSOCIATION.—*Organized in 1846.*

The next annual meeting will be held June 7th, 8th, 9th and 10th, 1887, in Chicago, Ill. Following are the officers:

President, E. H. Gregory, M.D., St. Louis, Mo.

Permanent Secretary, W. B. Atkinson, M.D., Philadelphia, Penn.

Assistant Secretary, J. Nevins Hyde, M.D., Chicago, Ill.

Treasurer, Richard J. Dunglison, M.D., Philadelphia, Penn.

Librarian, C. H. A. Kleinschmidt, M.D., Washington, D. C.

Chairman of Committee of Arrangements, Charles Gilman Smith, M.D., Chicago, Ill.

---

NINTH INTERNATIONAL MEDICAL CONGRESS — TRANS-ATLANTIC RATES.

The Chairman of the Committee of Arrangements at Washington reports that reliable arrangements have been made by which members wishing to attend the International Medical Congress in Washington, September 5, 1887, can be accommodated by the following steamship lines at the liberally reduced rates mentioned, viz.:

Red Star Line — $100, Antwerp to New York and return.

Inman Line—$100, Liverpool to New York and return.

Hamburg Line—$90, Hamburg to New York and return.

Royal Netherland—$80, Amsterdam to New York and return.

And that the several lines named have consented to extend the same rates to the families of members, as the following letter shows:

WASHINGTON, D. C., FEB. 14, 1887.

A. Y. P. GARNETT, M.D., *Chairman of the Committee of Arrangements of the International Medical Congress:*

*Dear Doctor*:—I am happy to inform you that, through the instrumentality of Mr. Edward F. Droop, agent in this city for the Lines, who has manifested so much interest in this matter, we have been able to secure from the Hamburg-American, the Red Star and the Inman Lines the offer of the same reductions for the families of members of the Congress as those I have already reported for the members themselves.

Very truly yours,

J. W. H. LOVEJOY,

Chairman Committee on Transportation.

In order to aid the work of the Committee on Transportation, the State Department at Washington has instructed the resident U. S. Consuls at European ports from which the steamships leave to actively aid in ascertaining the number of those wishing to avail themselves of the reduced rates offered. This is more fully explained by the two following letters:

FEB. 14, 1887.

*Editor of the Journal of the American Medical Association:*

*Dear Sir*:—The following additional information relative to transatlantic transportation is furnished for publication:

The White Star Line as well as the Cunard Line, having declined to make any reduction in fare, you will please strike out *Havre* from the list of European ports last published.

I enclose also, for publication, a copy of instructions which the State

Department has kindly sent the resident U. S. Consuls at the ports of Liverpool, Hamburg, Bremen and Antwerp.

Very respectfully,

A. Y. P. GARNETT, M.D.,

Chairman Committee of Arrangements for the International Medical Congress.

DEPARTMENT OF STATE, WASHINGTON, FEB. 5, 1887.

*Sir:*—The Committee of Arrangements of the International Medical Congress, which meets in Washington in September next, desire to ascertain as nearly as possible the number of delegates who will attend the same, with a view of making favorable terms for their transportation. For this purpose you are therefore instructed to bring the matters to the attention of those interested by such means as you deem best, requesting that you may be furnished with the names of such delegates as will attend, and the number of ladies who will accompany them. The result of your efforts should be promptly reported. I am, sir, your obedient servant,

J. D. PORTER,

Assistant Secretary.

### BOOKS RECEIVED.

---

*How we Treat Wounds To-day.* By Robert T. Morris, M.D. New York: G. P. Putnam's Sons. Chicago: A. C. McClurg & Company.

*A Treatise on the Principles and Practice of Medicine.* By Austin Flint, M.D., LL.D. Philadelphia: Lea Brothers & Company. Chicago: A. C McClurg & Company.

*Massage as a Mode of Treatment.* By William Murrell, M.D., F.R.C.P. Philadelphia: P. Blakiston, Son & Company. Chicago: W. T. Keener.

*Transactions of the American Surgical Association. Vol. IV.* Edited by J. Ewing Mears. M.D. Philadelphia: P. Blakiston, Son & Company. Chicago: W. T. Keener.

*The Curability of Insanity.* By Pliny Earle, A.M., M.D. Philadelphia: J. B. Lippincott & Company. Chicago: A. C. McClurg & Company.

*The Functions of the Brain.* By David Ferrier, M.D., LL.D. New York: G. Putnam's Sons. Chicago: A. C. McClurg & Company.

*A Laboratory Guide in Urinalysis and Toxicology.* By R. A. Witthaus, A.M., M.D. New York: Wm. Wood & Company. Chicago: W. T. Keener.

*Hand-book of Practical Medicine. Vol. III. Diseases of the Nerves Muscles and Skin.* By Dr. Hermann Eichhorst. New York: Wm. Wood & Company. Chicago: W. T. Keener.

*House-Plants as Sanitary Agents.* By J. M. Anders, M.D., Ph.D. Philadelphia: J. B. Lippincott & Company.

*A Manual of Obstetrics.* By A. F. A. King, M.D. Philadelphia: Lea Brothers & Company. Chicago: A. C. McClurg & Company.

*Outlines of the Pathology and Treatment of Syphilis and Allied Diseases.* By Hermann von Zeissl, M.D.; translated by H. Raphael, M.D.

*Manual of Operative Surgery.* By Joseph D. Bryant, M.D. New York: D. Appleton & Company. Chicago: A. C. McClurg & Company.

*A Text-book of Medicine, for Students and Practitioners.* By Dr. Adolf Strümpell. New York: D. Appleton & Company. Chicago: A. C. McClurg & Company.

*Special Pathological Anatomy and Pathogenesis.* By E. Ziegler. London and New York: MacMillan & Company. Chicago: W. T. Keener.

### REPORTS AND PAMPHLETS RECEIVED.

*Eighth Annual Report of the State Board of Health of Illinois.*

*Thirteenth Annual Report of the State Board of Health of Michigan.*

*Seventh Annual Report of the State Board of Health, Lunacy and Charities of Massachusetts.*

A Novel Procedure for the Removal of Sub-glottic Laryngeal Growths with a report of an illustrative case. *By Wm. Chapman Jarvis, M.D.*

Memorias, Leidas en la 2a Serie de Sessiones de la Sociedad Española de Laringologia, Otologia y Rinologia. 2a Fasiculo del Tomo I.

Des Cataractes et de leurs Opérations, Conférences, Cliniques Professés. *Par le Dr. Galezowski.*

Hard Chancre of the Eyelids and Conjunctiva. *By David DeBeck, M.D.*

General Atrophy of the Conducting Apparatus of the Ear. *By S. O. Richey, M.D.*

Naso-Pharyngeal Catarrh. *By J. G. Carpenter, M.D.*

A Case of Lithotomy with Artificial Opening for Drainage. *By J. G. Carpenter, M.D.*

Urethral Lithotomy in the Female. *By J. G. Carpenter, M.D.*

Method in Medical Study. *By Charles H. May, M.D.*

The Relation of the State and the Medical Profession. An Address. *By Charles J. Lundy, A.M., M.D.*

Report of the Surgeon-General of the Navy for the year 1885.

Circulars of Information of the Bureau of Education.

Surgical Notes from the Case-book of a General Practitioner. *By Wm. C. Wile, M.D.*

Report for the Year 1885–86. Presented by the Board of Managers of the Observatory, to the President and Fellows of Yale College.

Operations in the Drum-Head for Impaired Hearing; with fourteen cases. *By S. S. Bishop, M.D.*

Galvano-Cautery in Diseases of the Prostate, Bladder and Urethra. *By Robert Newman, M.D.*

Is Electrolysis a Failure in the Treatment of Urethral Stricture? *By Robert Newman, M.D.*

Medical Communications of the Massachusetts Medical Society. Vol. XIII. No. 5. 1886.

2cfk

SUBSCRIPTION PRICE, $3.00 PER YEAR, IN ADVANCE.

Whole Number, 327. MARCH, 1887. Vol. LIV. Number 3.

INDEX MEDICUS

LIBRARY INDEX S.G.O.

### THE CHICAGO MEDICAL JOURNAL AND EXAMINER

Editor: S. J. JONES, M.D., LL.D.

COLLABORATORS.

CHICAGO:

PROFESSOR E. ANDREWS, M.D., LL.D.

PROFESSOR JOHN BARTLETT, M.D.

PROFESSOR W. H. BYFORD, A.M., M.D.

PROFESSOR LESTER CURTIS, A.M., M.D.

PROFESSOR O. C. DEWOLF, A.M., M.D.

PROFESSOR E. C. DUDLEY, A.B., M.D.

PROFESSOR C. FENGER, M.D.

PROFESSOR J. N. HYDE, A.M., M.D.

PROFESSOR E. F. INGALS, A.M., M D.

PROFESSOR W. W. JAGGARD, A.M., M.D.

PROFESSOR F. S. JOHNSON, A.M., M.D.

PROFESSOR H. A. JOHNSON, M.D., LL.D.

PROFESSOR C. W. PURDY, M.D.

PROFESSOR CHARLES G. SMITH, A.M., M.D

PROFESSOR ELBERT WING, A.M., M.D.

MILWAUKEE, WISCONSIN:

PROFESSOR N. SENN, M.D.

WASHINGTON, D. C.:

S. O. RICHEY, M.D.

UNITED STATES ARMY:

SURGEON, D. L. HUNTINGTON, M.D.

UNITED STATES NAVY:

MEDICAL DIRECTOR, J. M. BROWNE, M.D.
MEDICAL DIRECTOR, A. L. GIHON, A.M., M.D.

